# Characterization and phylogenetic analysis of the complete chloroplast genome of *Saussurea sagittifolia* (Asteraceae, Cardueae)

**DOI:** 10.1080/23802359.2023.2281704

**Published:** 2023-11-17

**Authors:** Xiao-Feng Liu, Jun-Jia Luo, Ting-Yu Li, Hui Chen, Jia-yu Liu, Bo Li, Liang Dou, Zhi-Xi Fu

**Affiliations:** a Key Laboratory of Land Resources Evaluation and Monitoring in Southwest, Sichuan Normal University, Ministry of Education, Chengdu, China; b College of Life Sciences, Sichuan Normal University, Chengdu, China; c Nanba High School, Dazhou, China; d Sichuan Environmental Monitoring Center, Chengdu, China; e Museum of Natural History/School of Life Sciences, Key Laboratory of Bio-Resources and Eco-Environment of Ministry of Education, Key Laboratory of Conservation Biology on Endangered Wildlife of Sichuan Province, Sichuan University, Chengdu, China; f Sustainable Development Research Center of Resources and Environment of Western Sichuan, Sichuan Normal University, Chengdu, China

**Keywords:** *Saussurea sagittifolia*, complete chloroplast genome, phylogenetic

## Abstract

The species of *Saussurea sagittifolia* Y. S. Chen & S. R. Yi belongs to the family Asteraceae (Cardueae). The complete chloroplast genome of *S. sagittifolia* was assembled and annotated for the first time in this study. The complete chloroplast genome of *S. sagittifolia* was 152,535 bp, including a large single-copy (LSC) region of 83,511 bp, a small single-copy (SSC) region of 18,632 bp, and a pair of inverted repeats (IRs) of 25,196 bp. The overall GC content of the chloroplast genome was 37.7%. The chloroplast genome encoded 131 genes, including 87 protein-coding genes, 36 tRNA genes, and eight rRNA genes. Phylogenetic analysis based on complete chloroplast sequences revealed that it related closely to *Saussurea medusa*.

## Introduction

The *Saussurea* is one of the largest genera in the tribe Cardueae of family Asteraceae, with highly medicinal and ornamental value in China (Shi and Raab-Straube 2011; Li et al. 2022). It is mainly distributed in Asia, Europe, and North America (Shi and Raab-Straube 2011). *Saussurea sagittifolia* Y. S. Chen & S. R. Yi is a new species from the Bashan Mountains region in North Sichuan province, China in 2020 (Xu et al. 2020). The species of *S. sagittifolia* is close to *S. oligocephala* in morphological characteristics. However, it is different with the basal leaves triangular-ovate, a margin of small sharp teeth, apex acuminate, base with an auricular protrusion, and cauline leaves of more than 10, triangular, lanceolate, linear to subulate (Xu et al. 2020). In this study, we collected this species in Guangwu Shan Mountain, Bazhong City, Sichuan Province, China. We sequenced and reported the complete chloroplast genome of *S. sagittifolia* using next-generation sequencing technology for the first time. It will be helpful for well understanding the species delimitation studies and phylogenetic position of the genus *Saussurea* and *S. sagittifolia* in the Asteraceae.

## Materials and methods

### Sampling, extraction, and genome sequencing

**Figure 1.**
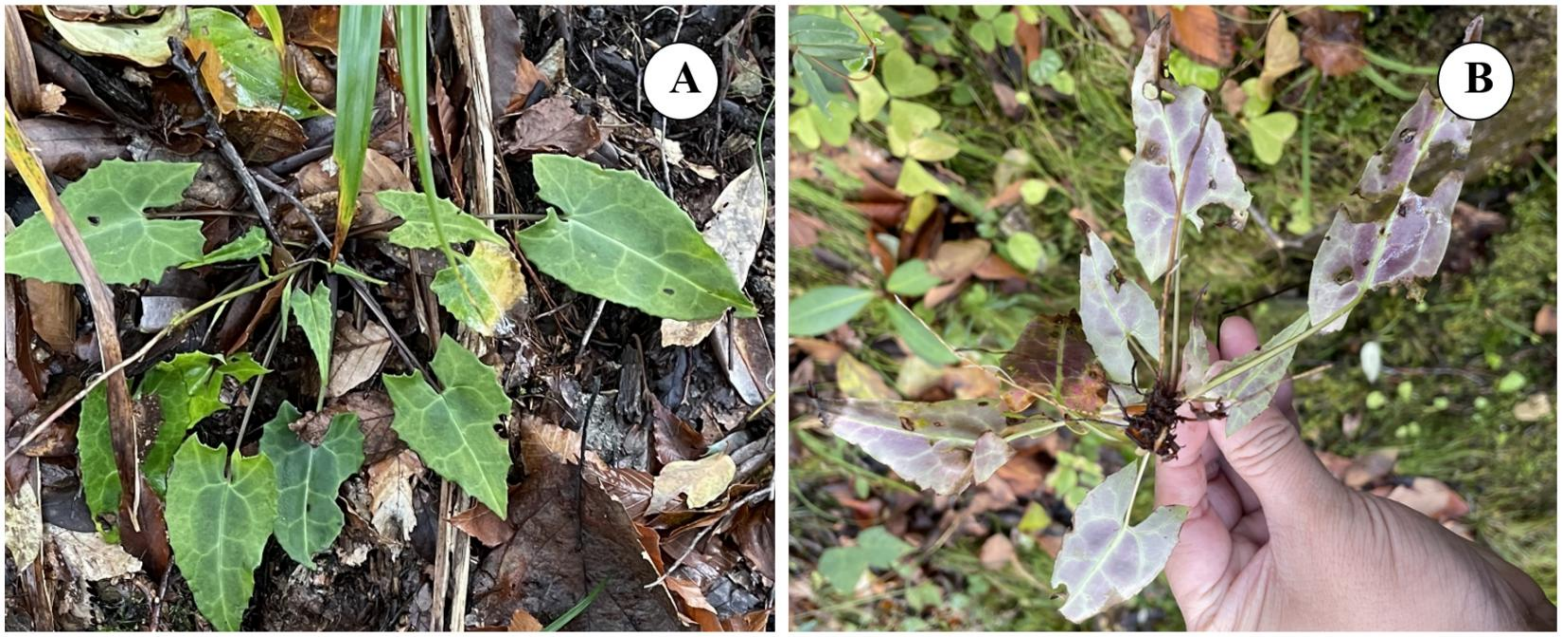
The morphology of leaf of *S. sagittifolia.* (a) The basal leaves of *S. sagittifolia.* (b) The leaves abaxially of *S. sagittifolia.* Leaf blades in middle and upper stem triangular, lanceolate, linear to subulate, apex long-caudate, base attenuate, margin with a few small sharp teeth to subentire. Leaves abaxially usually dark purple, capitula fewer, and involucres narrowly campanulate. The photos of *S. sagittifolia* were taken by the Zhi-Xi Fu in Guangwushan Mountain, Nanjiang county, Bazhong city, Sichuan province, China (the voucher number: Ya Deng, DY158).

The fresh leaves and specimen of *S. sagittifolia* (Figure 1) were collected from Guangwu Shan in Nanjiang County, Sichuan Province, China (106°48′3.16″E, 32°39′45.8″N). The voucher specimen was deposited at the Herbarium of Sichuan Normal University (SCNU) (Zhi-Xi Fu, fuzx2017@sicnu.edu.cn) under the voucher number: Ya Deng, DY158. Total genomic DNA was extracted using a modified CTAB method (Doyle and Doyle 1987). A paired-end library with an insert size of 150 bp was constructed, and the library was sequenced using Illumina NovaSeq 6000 platform at Beijing Genomics Institute (BGI, Shenzhen, China).

### Genome assembly and annotation

The high-quality reads were assembled with SPAdes v3.10.1 (Bankevich et al. 2012) and annotated by PGA (Qu et al. 2019) with default settings. To assess the accuracy of the assembly, we calculated the depth of coverage by mapping the reads to the chloroplast genome sequence using a specific protocol (https://doi.org/10.17504/protocols.io.4r3l27jkxg1y/v1). The annotation result was drawn using the CPGview program (http://www.1kmpg.cn/cpgview/) (Liu et al. 2023). The complete chloroplast sequence of *S. sagittifolia* deposited in GenBank of the National Center for Biotechnology Information (NCBI, https://www.ncbi.nlm.nih.gov, accession number ON094066).

### Phylogenetic analysis

In order to reveal the phylogenetic position of *S. sagittifolia* with other members of Asteraceae, a phylogenetic analysis was performed based on 24 complete chloroplast genomes of Asteraceae, and *Anthriscus cerefolium* (Apiaceae), *Kalopanax septemlobus* (Araliaceae) as outgroups. Sequence alignment was achieved using the MAFFT (Katoh and Standley 2013). A maximum-likelihood (ML) analysis was carried out with RaxML v7.2.8 based on the GTRGAMMA model on the CIPRES (https://www.phylo.org/) using 1000 bootstrap replicates (Stamatakis 2014).

## Results

### Genome structure analysis

**Figure 2.**
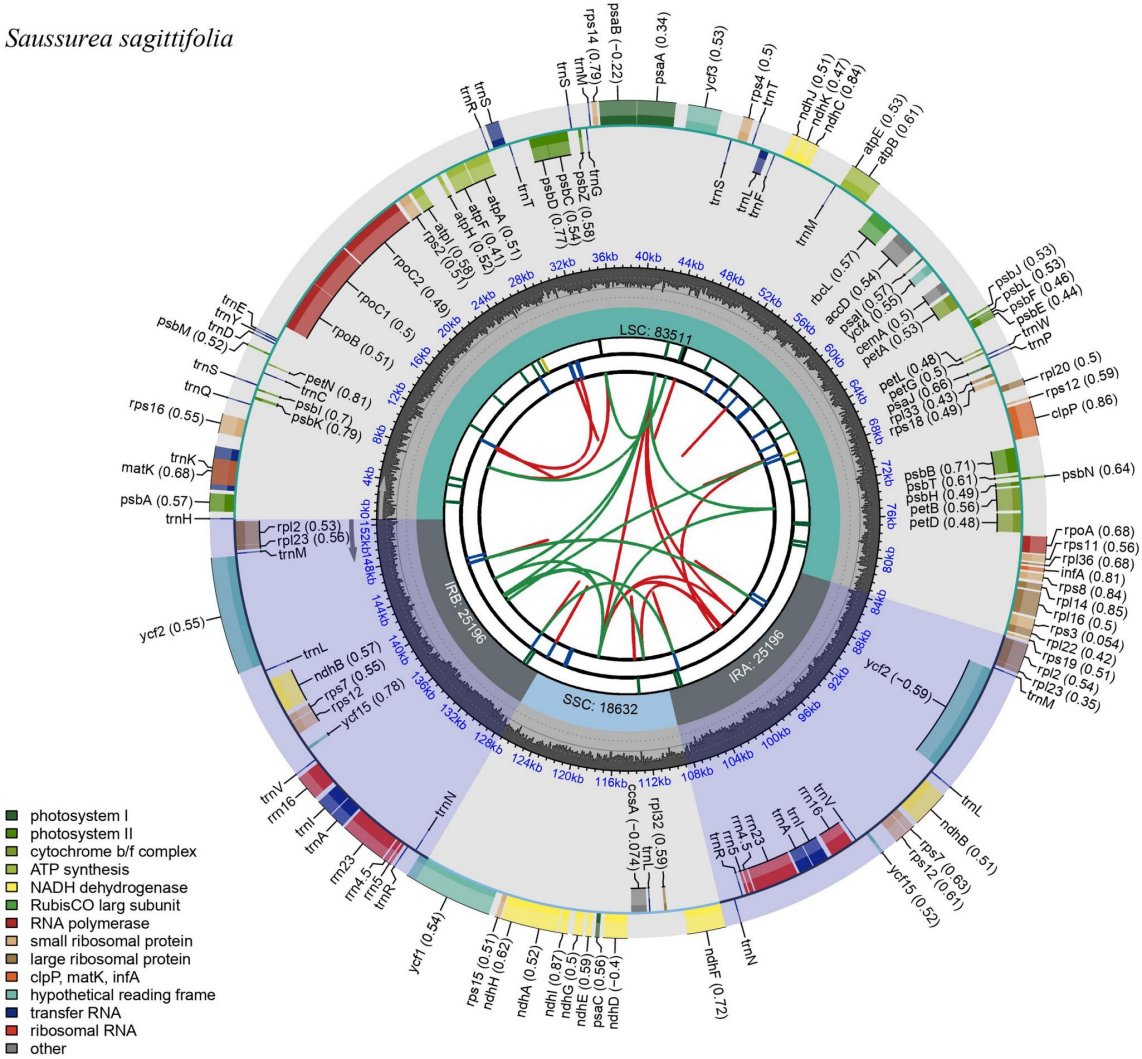
The chloroplast genome map of *S. sagittifolia* generated using CPGview. Boxes of different sizes and colors in the outermost circle represent genes and their lengths. Genes inside the circle are transcribed clockwise, and those on the outside are transcribed counter-clockwise. The grey area in the middle circle represents the variation of GC content at different positions, and the regions and lengths represented by the tetrameric structures (LSC, SSC, IRa, and IRb) are plotted in different colors on the inner circle.

The clean reads of *S. sagittifolia* were approximately 5.7 Gb. The complete chloroplast genome of *S. sagittifolia* was 152,535 bp in length, with an average depth of ×223.80 (Supplementary Figure 1). It had a typical quadripartite structure, including a large single-copy region (LSC, 83,511 bp), a small single-copy region (SSC, 18,632 bp), and two inverted repeat regions (IRs, 25,196 bp). The genome contained 131 genes, including 87 protein-coding genes, 36 tRNA genes, and eight rRNA genes (Figure 2). Most of the genes occurred in a single copy; however, seven protein-coding genes (*ndhB*, *rpl2*, *rpl23*, *rps12*, *rps7*, *ycf15*, and *ycf2*), eight tRNA genes (*trnA-UGC*, *trnI*, *trnL-CAA*, *trnM-CAU*, *trnN-GUU*, *trnR-ACG*, *trnS-UGA*, and *trnV-GAC*), and four rRNA genes (*4.5S*, *5S*, *16S*, and *23S*) were duplicated. In total, 16 genes contained introns, while *rps12*, *clpP*, and *ycf3* contained two introns. The *rps12* gene was a trans-spliced gene, with its 5′ end in the LSC region and its 3′ end positioned within the two IR regions (Supplementary Figure 2). Gene structure analysis was done for *rps16*, *rpoC1*, *atpF*, *ycf3*, *clpP*, *petB*, *petD*, *rpl16*, *rpl2*, *ndhB*, and *ndhA* difficult to annotate genes (Supplementary Figure 3). The overall GC content was 37.7%, whereas the corresponding values of LSC, SSC, and IR regions were 35.79%, 31.34%, and 43.12%, respectively.

### Phylogenetic analysis

**Figure 3.**
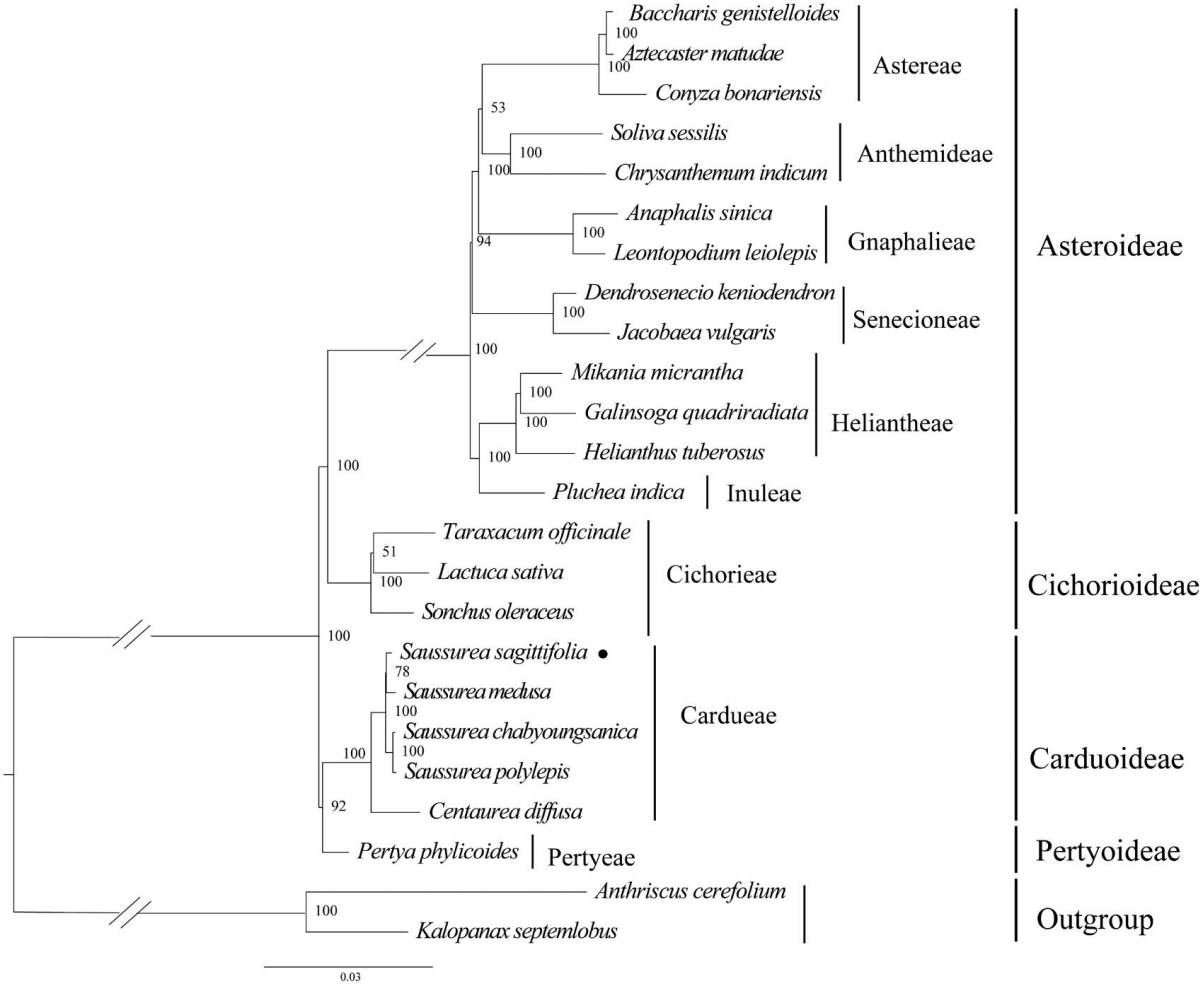
Maximum-likelihood phylogeny of *S. sagittifolia* and related taxa based on 24 complete chloroplast genomes. *Anthriscus cerefolium* (Apiaceae), *Kalopanax septemlobus* (Araliaceae) were used as outgroups. The maximum-likelihood bootstrap support values are along the branches. Circles represent newly sequenced species. The following sequences were used: *Aztecaster matudae* KX063935 (Vargas et al. 2017), *Baccharis genistelloides* KX063864 (Vargas et al. 2017), *Conyza bonariensis* KX792499 (Wang et al. 2018), *Chrysanthemum indicum* JN867589 (unpublished), *Soliva sessilis* KX063863 (Vargas et al. 2017), *Anaphalis sinica* KX148081 (unpublished), *Leontopodium leiolepis* KM267636 (unpublished), *Dendrosenecio keniodendron* KY434193 (unpublished), *Jacobaea vulgaris* HQ234669 (Doorduin et al. 2011), *Galinsoga quadriradiata* KX752097 (Wang et al. 2018), *Mikania micrantha* KX154571 (Huang et al. 2016), *Helianthus tuberosus* MG696658 (unpublished), *Pluchea indica* MG452144 (Zhang et al. 2017), *Taraxacum officinale* KU361241 (unpublished), *Lactuca sativa* AP007232 (unpublished), *Sonchus oleraceus* MG878405 (Hereward et al. 2018), *Saussurea sagittifolia* ON094066 (in this study), *Saussurea polylepis* MF695711 (Seon et al. 2017), *Centaurea diffusa* KJ690264 (unpublished), *Pertya phylicoides* MN935435 (Wang et al. 2020), *Anthriscus cerefolium* GU456628 (Downie and Jansen 2015), and *Kalopanax septemlobus* NC022814 (Li et al. 2013).

The phylogenetic analysis revealed that *S. sagittifolia* was belonging to the subfamily Carduoideae tribe Cardueae. Furthermore, *S. sagittifolia* was closely related to *S. medusa.* The genus *Saussurea* was recovered as a monophyletic group (Figure 3). The chloroplast genome sequence of *S. sagittifolia* in this study might provide vital information for phylogenetic and evolutionary studies in Asteraceae.

## Discussion and conclusions

In this study, the chloroplast genome sequence of *S. sagittifolia* was assembled and annotated for the first time. The genome size, organization, and structure were highly conserved and similar to other Asteraceae species (Zhang et al. 2019; Shahzadi et al. 2020; Yun and Kim 2022; Jin et al. 2023; Liu et al. 2023). The phylogenetic results indicated that *S. sagittifolia* exhibited the closest relationship with *S. polylepis*. This study provided new information for the phylogenetic relationship of the Asteraceae family.

## Supplementary Material

Supplemental Material

## Data Availability

The genome sequence data that support the findings of this study are openly available in GenBank of NCBI at https://www.ncbi.nlm.nih.gov/ under the accession no. ON094066. The associated BioProject, SRA, and Bio-Sample numbers are PRJNA939009, SRR23682417, and SAMN33558826, respectively.

## References

[CIT0001] Bankevich A, Nurk S, Antipov D, Gurevich AA, Dvorkin M, Kulikov AS, Lesin VM, Nikolenko SI, Pham S, Prjibelski AD, et al. 2012. SPAdes: a new genome assembly algorithm and its applications to single-cell sequencing. J Comput Biol. 19(5):455–477. doi: 10.1089/cmb.2012.0021. 22506599 PMC3342519

[CIT0002] Doorduin L, Gravendeel B, Lammers Y, Ariyurek Y, Chin-A-Woeng T, Vrieling K. 2011. The complete chloroplast genome of 17 individuals of pest species *Jacobaea vulgaris*: SNPs, microsatellites and barcoding markers for population and phylogenetic studies. DNA Res. 18(2):93–105. doi: 10.1093/dnares/dsr002. 21444340 PMC3077038

[CIT0003] Downie SR, Jansen RK. 2015. A comparative analysis of whole plastid genomes from the Apiales: expansion and contraction of the inverted repeat, mitochondrial to plastid transfer of DNA, and identification of highly divergent noncoding regions. Syst Bot. 40(1):336–351. doi: 10.1600/036364415X686620.

[CIT0004] Doyle JJ, Doyle JL. 1987. A rapid DNA isolation procedure for small quantities of fresh leaf tissue. Phytochem Bull. 19:11–15.

[CIT0005] Hereward JP, Werth JA, Thornby DF, Keenan M, Chauhan BS, Walter GH. 2018. Complete chloroplast genome of glyphosate resistant *Sonchus oleraceus* L. from Australia, with notes on the small single copy (SSC) region orientation. Mitochondrial DNA B Resour. 3(1):363–364. doi: 10.1080/23802359.2018.1450682. 33474170 PMC7799887

[CIT0006] Huang L, Wang Z, Wang T, Su YJ. 2016. The complete chloroplast genome sequence of *Mikania micrantha* (Asteraceae), a noxious invasive weed to South China. Mitochondrial DNA B Resour. 1(1):603–604. doi: 10.1080/23802359.2016.1209090. 33473567 PMC7800490

[CIT0007] Jin GZ, Li WJ, Song F, Yang L, Wen ZB, Feng Y. 2023. Comparative analysis of complete *Artemisia* subgenus *Seriphidium* (Asteraceae: Anthemideae) chloroplast genomes: insights into structural divergence and phylogenetic relationships. BMC Plant Biol. 23(1):136. doi: 10.1186/s12870-023-04113-1. 36899296 PMC9999589

[CIT0008] Katoh K, Standley DM. 2013. MAFFT multiple sequence alignment software version 7: improvements in performance and usability. Mol Biol Evol. 30(4):772–780. doi: 10.1093/molbev/mst010. 23329690 PMC3603318

[CIT0010] Li R, Ma PF, Wen J, Yi TS. 2013. Complete sequencing of five Araliaceae chloroplast genomes and the phylogenetic implications. PLOS One. 8(10):e78568. doi: 10.1371/journal.pone.0078568. 24205264 PMC3799623

[CIT0011] Li T, Xu LS, Chen YS. 2022. *Saussurea khunjerabensis* (Asteraceae, Cardueae), a new species from Pamir. Phytotaxa. 561(1):65–74. doi: 10.11646/phytotaxa.561.1.6.

[CIT0012] Liu S, Ni Y, Li J, Zhang X, Yang H, Chen H, Liu C. 2023. CPGView: a package for visualizing detailed chloroplast genome structures. Mol Ecol Resour. 23(3):694–704. doi: 10.1111/1755-0998.13729. 36587992

[CIT0013] Liu XF, Luo JJ, Zhang MK, Wang Q, Liu J, Wu D, Fu ZX. 2023. Phylogenomic analysis of two species of *Parasenecio* and comparative analysis within tribe Senecioneae (Asteraceae). Diversity. 15(4):563. doi: 10.3390/d15040563.

[CIT0014] Qu XJ, Moore MJ, Li DZ, Yi TS. 2019. PGA: a software package for rapid, accurate, and flexible batch annotation of plastomes. Plant Methods. 15(1):50. doi: 10.1186/s13007-019-0435-7. 31139240 PMC6528300

[CIT0015] Seon A, Yun SA, Gil HY, Kim SC. 2017. The complete chloroplast genome sequence of *Saussurea polylepis* (Asteraceae), a vulnerable endemic species of Korea. Mitochondrial DNA B Resour. 2(2):650–651. doi: 10.1080/23802359.2017.1375881. 33473934 PMC7799718

[CIT0016] Shahzadi I, Abdullah , Mehmood F, Ali Z, Ahmed I, Mirza B. 2020. Chloroplast genome sequences of *Artemisia maritima* and *Artemisia absinthium*: comparative analyses, mutational hotspots in genus *Artemisia* and phylogeny in family Asteraceae. Genomics. 112(2):1454–1463. doi: 10.1016/j.ygeno.2019.08.016. 31450007

[CIT0017] Shi Z, Raab-Straube EV. 2011. *Saussurea* Candolle. In: Wu ZY, Raven PH, Hong DY, editors. Flora of China. Vols. 20–21. Beijing; St. Louis: Science Press; Missouri Botanical Garden Press; p. 56–149.

[CIT0018] Stamatakis A. 2014. RAxML version 8: a tool for phylogenetic analysis and post-analysis of large phylogenies. Bioinformatics. 30(9):1312–1313. doi: 10.1093/bioinformatics/btu033. 24451623 PMC3998144

[CIT0019] Vargas OM, Ortiz EM, Simpson BB. 2017. Conflicting phylogenomic signals reveal a pattern of reticulate evolution in a recent high-Andean diversification (Asteraceae: Astereae: Diplostephium). New Phytol. 214(4):1736–1750. doi: 10.1111/nph.14530. 28333396

[CIT0020] Wang AS, Wu HW, Zhu XC, Lin JM. 2018. Species identification of *Conyza bonariensis* assisted by chloroplast genome sequencing. Front Genet. 9(374):374. doi: 10.3389/fgene.2018.00374. 30254661 PMC6141629

[CIT0021] Wang B, Zhao Q, Wang XH, Fu ZX. 2020. The complete chloroplast genome of *Pertya phylicoides* (Asteraceae, Pertyeae): a shurby endemic species from China. Mitochondrial DNA B Resour. 5(1):963–964. doi: 10.1080/23802359.2020.1722763. 33366828 PMC7748547

[CIT0022] Wang XY, Zhou ZS, Liu G, Qian ZQ. 2018. Characterization of the complete chloroplast genome of the invasive weed *Galinsoga quadriradiata* (Asterales: Asteraceae). Conserv Genet Resour. 10(1):89–92. doi: 10.1007/s12686-017-0771-8.

[CIT0023] Xu LS, Yi SR, Chen YS. 2020. *Saussurea sagittifolia* (Asteraceae, Cardueae), a new species from the Bashan Mountains region of China. Phytotaxa. 472(3):295–298. doi: 10.11646/phytotaxa.472.3.9.

[CIT0024] Yun S, Kim S-C. 2022. Comparative plastomes and phylogenetic analysis of seven Korean endemic *Saussurea* (Asteraceae). BMC Plant Biol. 22(1):550. doi: 10.1186/s12870-022-03946-6. 36443690 PMC9706989

[CIT0025] Zhang X, Deng T, Moore MJ, Ji YH, Lin N, Zhang HJ, Meng AP, Wang HC, Sun YX, Sun H. 2019. Plastome phylogenomics of *Saussurea* (Asteraceae: Cardueae). BMC Plant Biol. 19(1):290. doi: 10.1186/s12870-019-1896-6. 31266465 PMC6604455

[CIT0026] Zhang Y, Zhang J, Yang Y, Liu Q. 2017. Complete chloroplast genome of *Pluchea indica* (L.) Less. (Asteraceae) and its phylogenetic analysis. Mitochondrial DNA B Resour. 2(2):918–919. doi: 10.1080/23802359.2017.1413299. 33474035 PMC7800182

