# Supplementary material for: Characterization and phylogenetic analysis of the complete chloroplast genome of Saussurea sagittifolia (Asteraceae, Cardueae)
Source: Mitochondrial DNA B Resour. 2023 Nov 17;8(11):1268–72. doi: 10.1080/23802359.2023.2281704 (PMC10769533; doi:10.1080/23802359.2023.2281704)
Supplement: Supplemental Material [file TMDN_A_2281704_SM0356.docx]

**[Supplementary Figure 1](https://doi.org/10.1080/23802359.2023.2220426).** The overall coverage depth of *Saussurea sagittifolia* chloroplast genome assembly.

**
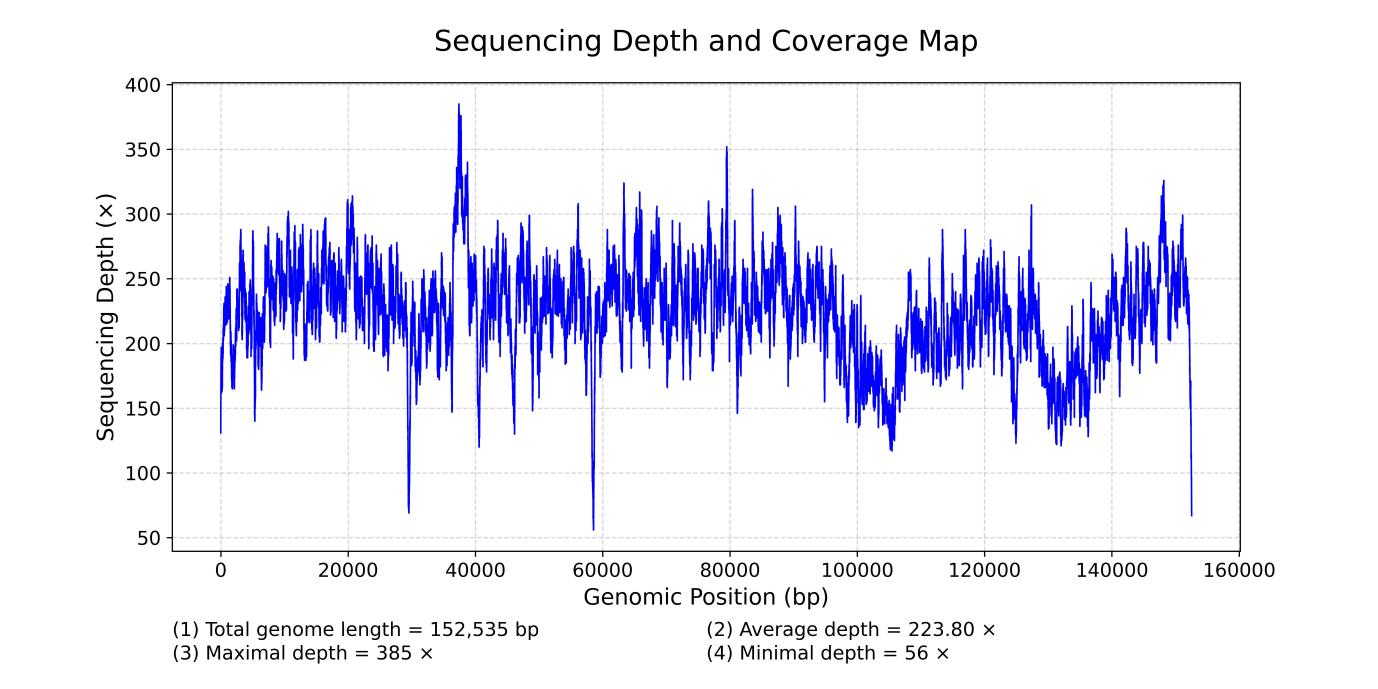
**

**[Supplementary Figure 2](https://doi.org/10.1080/23802359.2023.2220426).** Schematic map of the trans-splicing genes in the chloroplast genome of *Saussurea sagittifolia*.

**
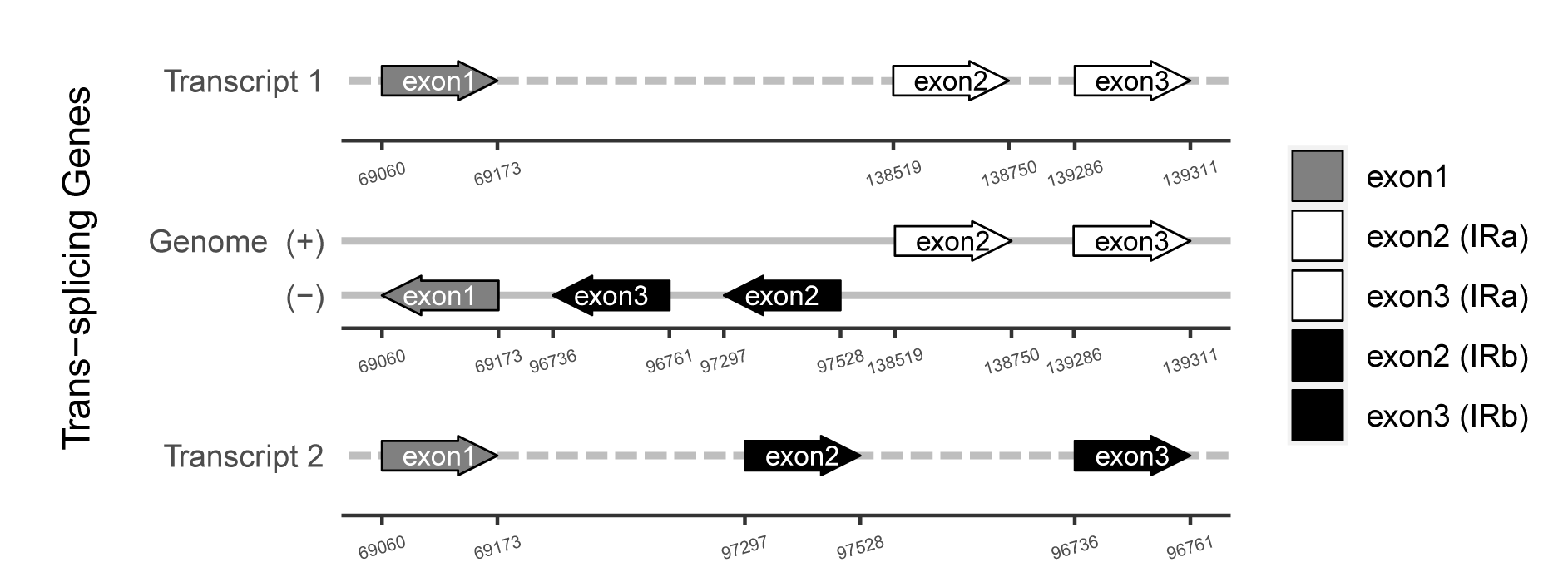
**

**[Supplementary Figure 3](https://doi.org/10.1080/23802359.2023.2220426).** Schematic map of the cis-splicing genes in the chloroplast genome of *Saussurea sagittifolia*.

**
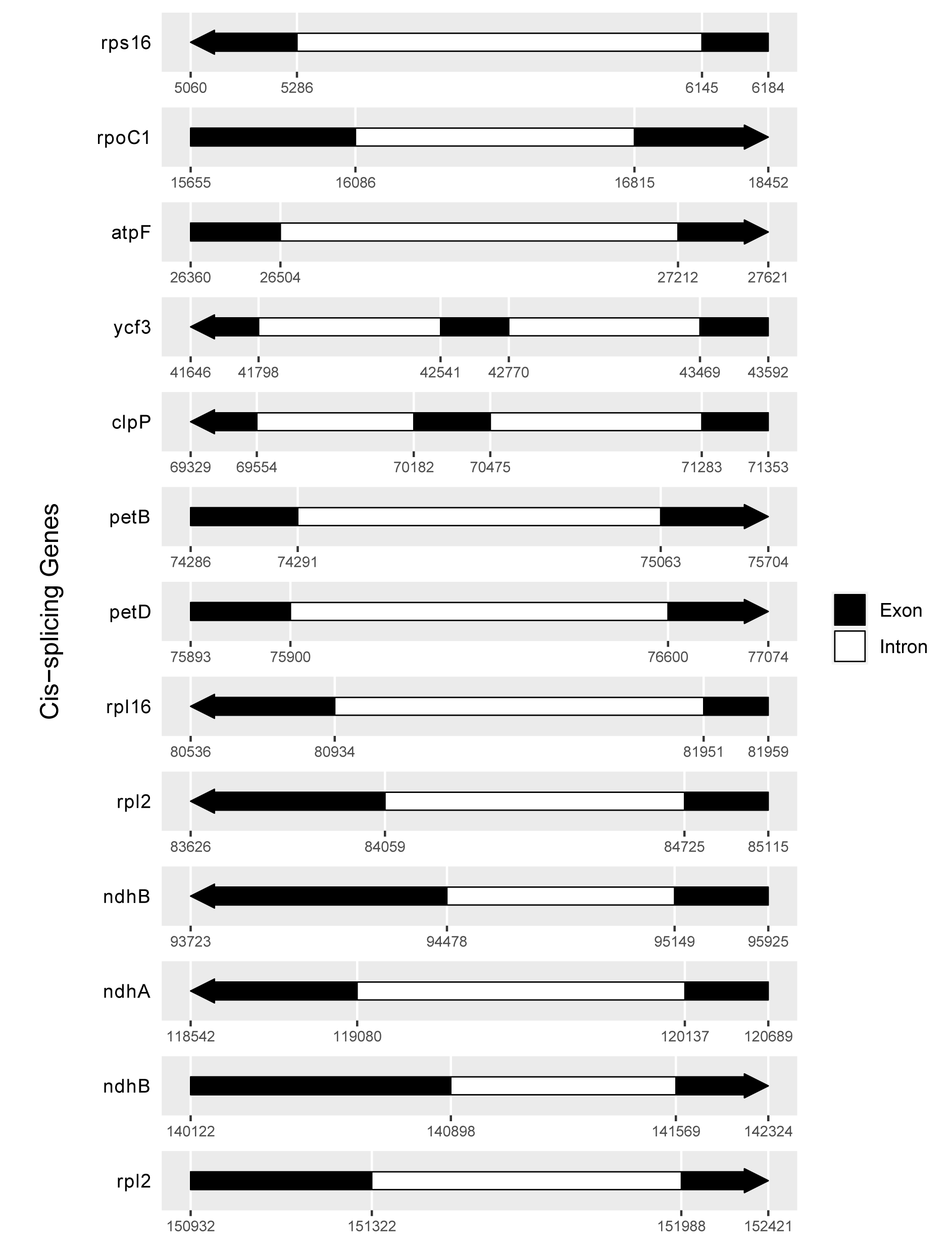
**
